# Supplementary material for: Two-Dimensional (2D) TM-Tetrahydroxyquinone Metal–Organic Framework for Selective CO2 Electrocatalysis: A DFT Investigation
Source: Nanomaterials (Basel). 2022 Nov 17;12(22):4049. doi: 10.3390/nano12224049 (PMC9696692; doi:10.3390/nano12224049)
Supplement: Supplementary file 1 [file nanomaterials-12-04049-s001.zip › nanomaterials-2030873-supplementary.pdf]

2D TM-Tetrahydroxyquinone Metal–Organic Framework for Selective CO_2 Electrocatalysis: A DFT Investigation

Supporting Information

Xianshi Zeng [1,2], Chuncai Xiao [3], Luliang Liao [2,3], Zongxing Tu [4], Zhangli Lai [1], Kai Xiong [5,6] and Yufeng Wen [1,*]

1 School of Mathematical Sciences and Physics, Jinggangshan University, Ji'an 343009, China

2 Institute for Advanced Study, Nanchang University, Nanchang 330031, China

3 School of Mechanical and Electrical Engineering, Xinyu University, Xinyu 338004, China

4 School of Chemistry, Nanchang University, Nanchang 330031, China

5 Materials Genome Institute, National Center for International Research on Photoelectric and Energy Materials, School of Materials and Energy, Yunnan University, Kunming 650091, China

6 Advanced Computing Center, Information Technology Center, Yunnan University, Kunming 650091, China.

* Correspondence: jgsuwyf@sina.com

Table S1 Lattice constant a and b of TM−THQ in Å, where TM are the metal atoms of the first transition metal series.

TM−THQ	Lattice(a)	Lattice(b)
Sc-THQ	13.962	13.928
Ti-THQ	13.651	13.690
V-THQ	13.499	13.480
Cr-THQ	13.369	13.367
Mn-THQ	13.247	13.268
Fe-THQ	13.154	13.175
Co-THQ	13.013	13.065
Ni-THQ	13.110	13.087
Cu-THQ	13.492	13.424
Zn-THQ	13.591	13.528

Table S2 E_c is the cohesive energy of the bulk TM, E_b is the binding energy between the TM and the TM–THQ, where TM are the metal atoms of the first transition metal series.

TM-THQ	E_b(eV)	E_c(eV)
Sc	-10.866	-4.423
Ti	-11.044	-5.956
V	-10.008	-6.578
Cr	-10.244	-4.011
Mn	-7.822	-3.761
Fe	-7.861	-5.264
Co	-7.831	-6.051
Ni	-7.076	-6.017
Cu	-5.403	-3.4313
Zn	-4.627	-1.054

Table S3 Gibbs free energy change (ΔG/eV) of the first protonation step in the CO_2 reduction reaction (CO_2RR) and H_2 evolution reaction (HER) on the TM–THQ

TM-THQ	**ΔG[*H]**	**ΔG*COOH**	**ΔG*OCHO**
Sc-THQ	0.984	2.136	-0.443
Ti-THQ	0.203	0.318	-0.535
V-THQ	0.055	0.120	-0.265
Cr-THQ	-0.087	-0.178	-0.285
Mn-THQ	-0.250	-0.353	-0.214
Fe-THQ	-0.195	-0.299	0.132
Co-THQ	0.329	0.241	0.793
Ni-THQ	0.913	1.0463	1.251
Cu-THQ	1.255	0.888	1.044
Zn-THQ	1.177	0.7833	0.307

Table S4 Gibbs free energy change for each protonation step of Sc-THQ electrocatalytic CO_2 reduction.

$n(H^++e^-)$transferred	Chemical reaction equation	ΔG
1	$* + CO_2 + H^+ + e^- \rightarrow$ *OCHO	-0.443
2	*OCHO+H++e-→*OCHOH	-0.281
3	*OCHOH+H++e-→*CHO+H2O	1.289
	*OCHOH+H++e-→*OCH+H2O	1.676
	OCHOH→+HCOOH	0.816

Table S5 Gibbs free energy change for each protonation step of Co-THQ electrocatalytic CO_2 reduction.

$n(H^++e^-)$transferred	Chemical reaction equation	ΔG
1	$* + CO_2 + H^+ + e^- \rightarrow *COOH$	0.241
2	$*COOH + H^+ + e^- \rightarrow *CO + H_2O$	0.178
3	$*CO + H_2O + H^+ + e^- \rightarrow *CHO + H_2O$	-0.476
	$*CO + H_2O + H^+ + e^- \rightarrow *COH + H_2O$	1.294
	$*CO + H_2O \rightarrow * + CO + H_2O$	0.528
4	$*CHO + H_2O + H^+ + e^- \rightarrow *OCH_2 + H_2O$	-0.130
5	$*OCH_2 + H_2O + H^+ + e^- \rightarrow *OCH_3 + H_2O$	0.532
	$*OCH_2 + H_2O + H^+ + e^- \rightarrow * + HCHO + H_2O + H^+ + e^-$	0.195

Table S6 Gibbs free energy change for each protonation step of Ti-THQ electrocatalytic CO_2 reduction.

$n(H^++e^-)$transferred	Chemical reaction equation	ΔG
1	$* + CO_2 + H^+ + e^- \rightarrow *OCHO$	-0.535
2	$*OCHO + H^+ + e- \rightarrow *OCHOH$	-0.156
3	$*OCHOH + H^+ + e^- \rightarrow *CHO + H_2O$	1.043
	$*OCHOH + H^+ + e^- \rightarrow *OCH + H_2O$	1.651
4	$*CHO + H_2O + H^+ + e^- \rightarrow *OCH_2 + H_2O$	-1.118
5	$*OCH_2 + H_2O + H^+ + e^- \rightarrow *OCH_3 + H_2O$	-0.729
6	$*OCH_3 + H_2O + H^+ + e^- \rightarrow *CH_3OH + H_2O$	-0.277
	$*OCH_3 + H_2O + H^+ + e^- \rightarrow *O + CH_4 + H_2O$	-0.249
7	$*CH_3OH + H_2O + H^+ + e^- \rightarrow *OH + CH_4 + H_2O$	-1.119
	$*O + CH_4 + H_2O + H^+ + e^- \rightarrow *OH + CH_4 + H_2O$	-1.147
8	$*OH + CH_4 + H_2O + H^+ + e^- \rightarrow * + CH_4 + 2H_2O$	0.473

Table S7 Gibbs free energy change for each protonation step of V-THQ electrocatalytic CO_2 reduction.

$n(H^+ + e^-)$transferred	Chemical reaction equation	ΔG
1	$* + CO_2 + H^+ + e^- \rightarrow *OCHO$	-0.265
2	$*OCHO + H^+ + e^- \rightarrow *OCHOH$	-0.457
3	$*OCHOH + H^+ + e^- \rightarrow *CHO + H_2O$	0.663
	$*OCHOH + H^+ + e^- \rightarrow *OCH + H_2O$	1.805
4	$*CHO + H_2O + H^+ + e^- \rightarrow *OCH_2 + H_2O$	-0.611
5	$*OCH_2 + H_2O + H^+ + e^- \rightarrow *OCH_3 + H_2O$	-0.663
6	$*OCH_3 + H_2O + H^+ + e^- \rightarrow *CH_3OH + H_2O$	-0.339
	$*OCH_3 + H_2O + H^+ + e^- \rightarrow *O + CH_4 + H_2O$	-0.487
7	$*O + CH_4 + H_2O + H^+ + e^- \rightarrow *OH + CH_4 + H_2O$	-0.835
7	$*CH_3OH + H_2O + H^+ + e^- \rightarrow *OH + CH_4 + H_2O$	-0.983
8	$*OH + CH_4 + H_2O + H^+ + e^- \rightarrow * + CH_4 + 2H_2O$	0.241

Table S8 Gibbs free energy change for each protonation step of Cr-THQ electrocatalytic CO_2 reduction.

$n(H^+ + e^-)$transferred	Chemical reaction equation	ΔG
1	$* + CO_2 + H^+ + e^- \rightarrow *COOH$	-0.178
1	$* + CO_2 + H^+ + e^- \rightarrow *OCHO$	-0.285
2	$*COOH + H^+ + e^- \rightarrow *CO + H_2O$	-0.556
2	$*OCHO + H^+ + e^- \rightarrow *OCHOH$	-0.491
3	$*CO + H_2O + H^+ + e^- \rightarrow *CHO + H_2O$	0.211
	$*CO + H_2O + H^+ + e^- \rightarrow *COH + H_2O$	1.487
	$*OCHOH + H^+ + e^- \rightarrow *CHO + H_2O$	0.253
	$*OCHOH + H^+ + e^- \rightarrow *OCH + H_2O$	1.602
4	$*CHO + H_2O + H^+ + e^- \rightarrow *OCH_2 + H_2O$	-0.371
5	$*OCH_2 + H_2O + H^+ + e^- \rightarrow *OCH_3 + H_2O$	-0.493
6	$*OCH_3 + H_2O + H^+ + e^- \rightarrow *CH_3OH + H_2O$	-0.498
	$*OCH_3 + H_2O + H^+ + e^- \rightarrow *O + CH_4 + H_2O$	-0.455
7	$*O + CH_4 + H_2O + H^+ + e^- \rightarrow *OH + CH_4 + H_2O$	-0.944
	$*CH_3OH + H_2O + H^+ + e^- \rightarrow *OH + CH_4 + H_2O$	-0.901
8	$*OH + CH_4 + H_2O + H^+ + e^- \rightarrow * + CH_4 + 2H_2O$	0.032

Table S9 Gibbs free energy change for each protonation step of Mn-THQ electrocatalytic CO_2 reduction.

n(H^++e^-)transferred	Chemical reaction equation	ΔG
1	* + CO_2 + H^+ + e^- → *COOH	-0.353
2	*COOH + H^+ + e^- → *CO + H_2O	-0.478
3	*CO + H_2O + H^+ + e^- → *CHO + H_2O	0.145
	*CO + H_2O + H^+ + e^-→*COH + H_2O	1.585
4	*CHO + H_2O + H^+ + e^- → *OCH_2 + H_2O	-0.171
5	*OCH_2 + H_2O + H^+ + e^- →*OCH_3 + H_2O	-0.344
	*OCH_2 + H_2O + H^+ + e^- → * + HCHO + H_2O + H^++e^-	1.104
6	*OCH_3 + H_2O + H^++e^-→*CH_3OH + H_2O	-0.637
	*OCH_3 + H_2O + H^++ e^- → *O + CH_4+ H_2O	0.349
7	*CH_3OH + H_2O + H^+ + e^-→*OH + CH_4 + H_2O	-0.259
8	*OH + CH_4 + H_2O + H^+ + e^- → * + CH_4 + 2H_2O	-0.068

Table S10 Gibbs free energy change for each protonation step of Fe-THQ electrocatalytic CO_2 reduction.

n(H^++e^-)transferred	Chemical reaction equation	ΔG
1	* + CO_2 +H^+ + e^- → *COOH	-0.299
2	*COOH + H^+ + e^- → *CO + H_2O	-0.161
3	*CO + H_2O + H^+ + e^- → *CHO + H_2O	-0.141
	*CO+ H_2O + H^+ + e^- → *COH + H_2O	1.641
4	*CHO + H_2O + H^+ + e^-→ *OCH_2 + H_2O	0.147
5	*OCH_2 + H_2O + H^+ + e^- → *OCH_3 + H_2O	-0.166
	*OCH_2 + H_2O + H^+ + e^-→ * + HCHO + H2O + H^+ + e^-	0.715
6	*OCH_3 + H_2O + H^+ + e^- → *CH_3OH + H_2O	-0.881
	*OCH3 + H_2O + H^+ + e^- → *O + CH_4 + H_2O	0.558
7	*CH_3OH + H_2O + H^+ + e^- → *OH + CH_4 + H_2O	-0.125
8	*OH + CH_4+ H_2O + H^++ e^-→ * + CH_4+ 2H_2O	-0.345

Table S11 Gibbs free energy change for each protonation step of Zn-THQ electrocatalytic CO_2 reduction.

n(H^++e^-)transferred	Chemical reaction equation	ΔG
1	* + CO_2 + H^+ + e^-→ *COOH	0.783
	* + CO_2 + H^+ + e^-→ *OCHO	0.307
2	*COOH + H^+ + e^-→*CO+H_2O	-0.809
	*OCHO + H^+ + e^- → *OCHOH	-1.259
3	*CO + H_2O + H^+ + e^-→*CHO+H_2O	0.565
	*CO + H_2O + H^+ + e^- → *COH + H_2O	2.078
	*OCHOH + H^+ + e^-→ *CHO + H_2O	1.492
	*OCHOH + H^+ + e^- → *OCH + H_2O	1.844
	*CO + H_2O → * + CO + H_2O	0.971
	*OCHOH → * + HCOOH	0.841
4	*CHO + H_2O + H^+ + e^- → *OCH_2 + H_2O	-1.226
5	*OCH_2 + H_2O + H^+ + e^-→ *OCH_3 + H_2O	0.447
	*OCH_2 + H_2O + H^+ + e^- → * + HCHO + H_2O + H^+ + e^-	1.042
6	*OCH_3 + H_2O + H^+ + e^- → *CH_3OH + H_2O	-1.476
	*OCH_3 + H_2O + H^+ + e^- → *O + CH_4 + H_2O	1.656
7	*CH_3OH + H_2O + H^++ e^-→ *OH + CH_4 + H_2O	0.568
8	*OH + CH_4 + H_2O + H^+ + e^-→ * + CH_4 + 2H_2O	-0.949

Table S12 Gibbs free energy change for each protonation step of Cu-THQ electrocatalytic CO_2 reduction.

n(H^++e^-)transferred	Chemical reaction equation	ΔG
1	* + CO_2 + H^+ + e^-→ *COOH	0.888
	* + CO_2 + H^++ e^-→ *OCHO	1.044
2	*COOH + H^+ + e^-→ *CO + H_2O	-0.624
3	*CO + H_2O + H^+ + e^-→ *CHO +H_2O	0.767
	*CO + H_2O → * + CO + H_2O	0.652
	*CO + H_2O + H^+ + e^-→ *COH + H_2O	1.897
